# Evaluating implementation of a community-focused patient navigation intervention at an NCI-designated cancer center using RE-AIM

**DOI:** 10.1186/s12913-024-10919-y

**Published:** 2024-04-29

**Authors:** Elizabeth S. Ver Hoeve, Elizabeth Calhoun, Monica Hernandez, Elizabeth High, Julie S. Armin, Leila Ali-Akbarian, Michael Frithsen, Wendy Andrews, Heidi A. Hamann

**Affiliations:** 1 University of Arizona, Tucson, AZ USA; 2 Banner Health, Tucson, AZ USA; 3 University of Illinois Chicago, Chicago, IL USA; 4 Banyan Integrative Health, Tucson, AZ USA; 5 University of Arizona College of Medicine, Tucson, AZ USA

**Keywords:** Community-focused patient navigation, RE-AIM, Implementation science, Cancer care coordination, Sustainability, Supportive care interventions

## Abstract

**Background:**

Patient navigation is an evidence-based intervention that reduces cancer health disparities by directly addressing the barriers to care for underserved patients with cancer. Variability in design and integration of patient navigation programs within cancer care settings has limited this intervention’s utility. The implementation science evaluation framework, RE-AIM, allows quantitative and qualitative examination of effective implementation of patient navigation programs into cancer care settings.

**Methods:**

The Reach, Effectiveness, Adoption, Implementation, and Maintenance (RE-AIM) framework was used to evaluate implementation of a community-focused patient navigation intervention at an NCI-designated cancer center between June 2018 and October 2021. Using a 3-month longitudinal, non-comparative measurement period, univariate and bivariate analyses were conducted to examine associations between participant-level demographics and primary (i.e., barrier reduction) and secondary (i.e., patient-reported outcomes) effectiveness outcomes. Mixed methods analyses were used to examine adoption and delivery of the intervention into the cancer center setting. Process-level analyses were used to evaluate maintenance of the intervention.

**Results:**

Participants (*n* = 311) represented a largely underserved population, as defined by the National Cancer Institute, with the majority identifying as Hispanic/Latino, having a household income of $35,000 or less, and being enrolled in Medicaid. Participants were diagnosed with a variety of cancer types and most had advanced staged cancers. Pre-post-intervention analyses indicated significant reduction from pre-intervention assessments in the average number of reported barriers, F(1, 207) = 117.62, *p* < .001, as well as significant increases in patient-reported physical health, *t*(205) = − 6.004, *p* < .001, mental health, *t*(205) = − 3.810, *p* < .001, self-efficacy, *t*(205) = − 5.321, *p* < .001, and satisfaction with medical team communication, *t*(206) = − 2.03, *p* = .029. Referral patterns and qualitative data supported increased adoption and integration of the intervention into the target setting, and consistent intervention delivery metrics suggested high fidelity to intervention delivery over time. Process-level data outlined a successful transition from a grant-funded community-focused patient navigation intervention to an institution-funded program.

**Conclusions:**

This study utilized the implementation science evaluation framework, RE-AIM, to evaluate implementation of a community-focused patient navigation program. Our analyses indicate successful implementation within a cancer care setting and provide a potential guide for other oncology settings who may be interested in implementing community-focused patient navigation programs.

## Contributions to the literature

• This manuscript represents one of only a couple of manuscripts that have applied the Implementation Science Evaluation Framework, RE-AIM, to assess implementation of a patient navigation intervention within a cancer care setting.

• Operationalization of RE-AIM for evaluation of the patient navigation intervention implementation was comprehensive and closely aligned with guidance from www.re-aim.org and the RE-AIM Model Dimension Items Checklist developed by the National Cancer Institute in partnership with RE-AIM authors.

• Patient navigation is an evidence-based healthcare intervention that could benefit from guidance on effective intervention implementation effectiveness.

• Implementation evaluation metrics provided by RE-AIM support effective implementation of this evidence-based intervention into cancer care and suggest improvement in cancer health equity.

## Background

Patient navigation is an evidence-based intervention designed to reduce patients’ barriers to cancer care, strengthen patients’ adherence to cancer screening guidelines and treatment, and improve timeliness to cancer diagnostic resolution [1–13]. Accumulating evidence suggests that these explicated outcomes of patient navigation programs reduce cancer health disparities by reaching underserved patients directly to address their barriers to cancer care and coordination [13–15]. Despite widespread introduction of patient navigation programs into cancer centers throughout the United States over the last 5–10 years, national statistics on cancer health disparities remain stagnant [16, 17] and limitted data exist on the implementation effectiveness of these programs within cancer care settings [18–20]. Further, the majority of patient navigation programs have focused on early detection within several common cancer types (e.g., breast, colorectal, cervical), and knowledge about implementation of patient navigation programs inclusive of patients across the cancer care continuum and with diverse cancer types remains limited [21].

With the goal of eliminating health care disparities by improving cancer care access and coordination, the Commisssion on Cancer (CoC) required all accredited cancer centers to include patient navigation as part of their cancer care programing [22]. Although this CoC standard increased adoption of patient navigation programs across the country [23], these programs have varied substantially in design and scope [24]. Results from a recent nationwide survey suggest that nurses, social workers, and nonclinical staff currently provide patient navigation services but differ greatly in their roles and responsibilites based on clinical designation [25]. Additionally, patient navigation services differ by funding type and cancer care continuum stage, with nonclinical (i.e., lay) navigators being more likely to be grant-funded and more likely to be providing navigation services at earlier stages of the cancer care continuum [26].

Although patient navigation programs should not need to, nor be expected to, subscribe to a one-size-fits-all model because they are designed to accommodate and address the unique unmet needs of specific cancer care settings and patient populations [18, 24, 27], such variability in design, training, and integration of patient navigation programs into cancer care settings has presented challenges for standardized evaluations of patient navigation intervention implementation effectiveness [18, 19, 28]. Patient navigation was originally developed as an intervention to reduce cancer health disparities [14], and it has been shown to demonstrate strong efficacy among underserved patients with cancer [13, 29, 30]. Therefore, greater attention to implementation and sustainability of both the structural processes and the health equity processes associated with delivery of this evidence-based intervention is warranted [31].

This paper employs an implementation science evaluation approach [32] to assess the processes involved in effectively introducing and maintaining a community-focused patient navigation program at an NCI-designated cancer center that has a clinical affiliation with a nonprofit health care system [33] and is located in the Southwestern United States. Consistent with recent calls to utilize implementation science as a method for better addressing health equity within the context of intervention implementation evaluation [34], the development and evaluation of this community-focused patient navigation intervention’s implementation was guided by a health equity lens such that we utilized RE-AIM to guide our primary enrollment and evaluation objective to serve a medically underserved patient population. We evaluated our patient navigation program’s implementation during the period of June 2018 to October 2021, based on key domains of *Reach* (the representativeness of individuals participating in the intervention), *Effectiveness* (the impact of the intervention on specified outcomes), *Adoption* (the degree to which individuals within the setting utilize the intervention), *Implementation* (the consistency with which the intervention is delivered), and *Maintenance* (the extent to which the intervention becomes sustainable) [35]. To deliver maximum impact, optimal implementation research must explicitly examine both the individual-level impact of reaching primarily medically underserved patients to address barriers to care and the setting-level factors that promote long-term sustainability of patient navigation interventions. The present work describes the results of a 5-year effort to implement a community-focused patient navigation intervention into one cancer care setting using an operationalized RE-AIM framework to guide program implementation evaluation.

## Method

### Study design

As part of this intervention, a lay navigator (i.e., an individual with no higher education health care degree) was hired by the research team in 2018 and trained to become a community-focused patient navigator according to best practices [36]. Consistent with data collected through an earlier needs assessment at the cancer center [37], the investigator team was interested in hiring an interventionist who was fluent in English and Spanish, had strong connections to and engagement with the local community, was familiar with a local federally qualified health center, and who was exceptionally organized and motivated to help patients with cancer. The hired interventionist (i.e., navigator) was bilingual (English and Spanish) and bicultural (United States and Mexico). The same community-focused patient navigator remained active at 1.0 FTE throughout the duration of this intervention’s implementation. A supplemental interventionist was hired at .5 FTE for approximately one-year (2018–2019) to support intervention initiation.

The patient navigator worked individually with each patient who was referred to the community-focused patient navigation intervention. Over the course of a 3-month longitudinal non-comparative (i.e., continuous enrollment, no control group) measurement period, the patient navigator helped patients identify their barriers to cancer care, provided necessary services including referrals, community resource assistance, insurance-related assistance, and clinical care coordination improvements in an effort to reduce patients’ specific barriers, and at the end of the intervention, encouraged participants to re-assess their barriers to care. All aspects of the intervention, including all participant encounters as well as all efforts made on behalf of each participant, were systematically documented within REDCap by the navigator and study team personnel. Notably, the community-focused patient navigator was able to continue working with participants who expressed ongoing need for assistance following the conclusion of the intervention, although no additional data were collected beyond the 3-month follow-up.

Data collected for the intervention included: 1) demographic and disease characteristics, 2) pre- and post-barrier assessments conducted by the community-focused navigator as well as calculation of a post-intervention barrier resolution assessment conducted by two-trained research study members, and 3) patient-reported outcome questionnaires that were administered at intervention enrollment and again 3-months post-enrollment. Although enrolled participants were contacted approximately 2 months after enrollment by the navigator to review barrier resolution efforts (“two-month barrier assessment”), this assessment was primarily included to support implementation standardization and adherence, as opposed to data collection. The community-focused patient navigation intervention was initially implemented in June 2018 and enrolled its final patient in October 2021.

### Participant eligibility and recruitment

Any individual with a diagnosis of cancer, who had established cancer care in the clinical care system was eligible for participation in the community-focused patient navigation intervention. Specifically, participation was inclusive of any cancer type, cancer stage, or treatment point along the cancer care continuum (diagnosis, treatment, post-treatment survivorship). Although there were no eligibility criteria in terms of patient age, race, ethnicity, or primary language status, the community-focused navigation program was focused on enrolling underserved patients, and these patients often received referral priority from one of the referring clinical care teams (e.g., social worker, nurse, doctor, research coordinator, financial advisor). Once a referral was received, the navigator contacted the patient directly to introduce the study and obtain informed consent. This study received ethical approval from the Institutional Review Board at the University of Arizona (#1804483104), and informed consent was obtained from all participants. All methods were carried out in accordance with Declaration of Helsinki.

### Patient demographics and disease history

To characterize the sample of participants in the patient navigation intervention, patient-reported demographic histories were collected including ethnicity, race, primary language, gender, age, birth country, marital status, zip code, employment, highest level of education, household income, insurance, insurance type, home ownership, and housing insecurity. Consistent with the definition provided by the NCI, a demographic category labeled “underserved” was developed to represent any enrolled participant who was an ethnic/racial minority and/or insured through Medicaid [38]. Cancer history, including type of cancer, stage at diagnosis, and status on the cancer care continuum were collected via electronic medical records review by a trained study team member.

### Patient-reported barriers to Cancer care

At the time of intervention enrollment, the community-focused patient navigator conducted a barriers assessment to identify each patient’s specific barriers to cancer care. The barriers assessment was based on that used in the NCI Patient Navigation Research Program [39] and contained 88 possible barriers to cancer care. Identified patient-reported barriers to cancer care were documented, for each patient, by the community-focused patient navigator in REDCap.

At the 3-month post-intervention time point, all efforts taken by the patient navigator to resolve each participant’s barriers to cancer care (as documented in REDCap) were systematically reviewed and evaluated by two trained research team members. Reviewers used three categories to assess whether each barrier was: Not Addressed (i.e., the navigator either did not attempt to work on a solution to the barrier or attempted but was never able to identify a solution such as a referral or community resource); Addressed (i.e., the navigator was able to provide the patient with a solution such as a referral or a resource for a specific barrier); or Completely Addressed (i.e., the navigator was able to provide the patient with a solution such as a referral or a resource for a specific barrier and documentation within REDCap indicated that the patient was no longer experiencing the particular barrier). A ‘percent barrier addressed’ assessment was calculated for each patient using the formula: (#Addressed + #Completely Resolved)/(Total # Pre-Intervention Barriers) × 100.

### Patient-reported outcomes

#### Global Health

PROMIS Global Health (v1.2) (PROMIS Health Organization (PHO), 2018) is a health-related quality of life assessment that is part of the larger set of PROMIS (Patient-Reported Outcomes Measurement Information System) instruments [40, 41] funded by the National Institutes of Health (NIH) and normalized to the U.S. adult population. PROMIS Global Health assesses an individual’s physical, mental, and social health, and consists of two primary subscales: Global Physical Health (4 items: Global03, Global06, Global07rc, and Global08r) and Global Mental Health (4 items: Global02, Gloabl04, Global05, and Global10r). Raw scores were calculated based on the PROMIS Scoring Manual and then converted to T-scores using the PROMIS T-Score Tables [42]. Each item was rated on a 5-point Likert scale, where higher scores indicated that a greater amount of that subscale domain was being measured.

#### Self-efficacy

PROMIS General Self-Efficacy-Short Form 4a v1.0 [43] is a patient-reported assessment of one’s ability to successfully perform specific tasks or behaviors. This assessment contains four items that ask participants to rate, on a 5-point Likert scale, their levels of self-confidence in performing various tasks, where higher scores indicate a higher level of self-efficacy.

#### Patient satisfaction with medical services

Patient-reported satisfaction with medical services was evaluated using the *Patient Satisfaction Questionnaire-18 (PSQ-18*) [44] which contains 18 items organized into seven subscale domains including 1) general satisfaction, 2) technical quality, 3) interpersonal manner, 4) communication, 5) financial aspects, 6) time spent with doctor, and 7) accessibility and convenience. Each item was rated with a 5-point Likert response of strongly agree, agree, uncertain, disagree, and strongly disagree, where Strongly Agree (i.e., ‘5’) indicated higher patient-reported satisfaction with their medical services.

#### Patient satisfaction with patient navigation program

Patient-reported satisfaction with the patient navigator was evaluated using the *Patient Satisfaction with Navigator Interpersonal Relationship (PSN-I)* questionnaire [45]. Nine items assessed the extent to which the navigator spent sufficient time with the patient, made the patient feel comfortable, was dependable, was respectful of the patient, listened to the patient, was easy to communicate with, cared about the patient’s well-being, worked to problem-solve patient’s barriers to care, and was readily accessible. Each item was assessed with a 5-point Likert scale in which higher PSN-I total scores indicated higher patient-reported satisfaction with their interpersonal relationship with the patient navigator.

### Data analyses using RE-AIM framework

The RE-AIM framework dimensions, definitions, operationalizations, and data sources used in the evaluation of the implementation of this patient navigation intervention are outlined in Table 1 and are briefly described below. RE-AIM measurement is closely aligned with the RE-AIM Model Dimension Items Checklist [46], and data are presented in a manner consistent with the recommendations designed to systematically evaluate each dimension of the intervention.

**Table 1:** RE-AIM definitions, operationalizations, and data sources used in community-focused patient navigation intervention

RE-AIM Definitions	Operationalization	Data Source
**Reach**		
The absolute number, proportion, and representativeness of individuals who participated in the intervention.	• % of ‘underserved’ patients receiving cancer care at institution • % of ‘underserved’ patients enrolled in intervention • Demographic and disease characterizations • Intervention attrition and differential rates	• EMR • REDCap survey • Cancer Registry
**Effectiveness**		
The impact of an intervention on outcomes that demonstrate intervention effectiveness and broader outcomes of quality of life, satisfaction, and self-efficacy.	• Pre- and Post-Intervention Barriers • Patient-reported outcomes (PROMIS Global Health, PROMIS Self-Efficacy, PSQ-18, PSN-I) • Demographic and disease characterizations	• REDCap barrier tracking logs • REDCap survey
**Adoption**		
At staff-level, characterization of participating providers.	• Number of referrals over time • Qualitative assessment	• REDCap referral log • Post-intervention provider survey
**Implementation**		
At the setting level, assessment of fidelity to the intervention delivery, cost of the intervention, and adaptations made over time.	• % of patients who were contacted within 3 days of initial referral and % contacted for ‘2-month Check-in Call’ • Process-level description of REDCap database use, intervention cost, and adaptations	• REDCap screening and intervention database • Summary data from investigator meetings
**Maintenance**		
The extent to which the intervention becomes institutionalized or part of routine organizational practice and policy	• Program evaluation at 6 months post-grant funding • Process-level discussion of intervention’s transition from grant to institution-funding	• Summary data from investigator meetings

#### RE-AIM: reach (R)

The ‘Reach’ metric assessed the extent to which the patients who participated in the community-focused patient navigation intervention were representative of the population of medically underserved patients within the designated catchment area of the cancer center [47, 48]. Demographic and disease characteristics of enrolled participants were further analyzed to ensure overall comparability among participants who (a) consented to the intervention (*n* = 311), (b) completed the 3-month intervention (*n* = 255), and (c) completed the 3-month intervention as well as the post-intervention survey (*n* = 207). Descriptive statistics were used to assess the representativeness of the participant sample of the intervention in terms of (a) the number of individuals who agreed to participate in the community-focused patient navigation intervention relative to the total number of patients with cancer seen for clinical care, and (b) the extent to which the patients who participated in the community-focused patient navigation intervention were representative of the population of underserved patients within the designated catchment area of the cancer center. Consistent with the definition provided by the NCI, “underserved” patients were identified as individuals representing an ethnic/racial minority and/or insured through Medicaid [38].

#### RE-AIM: effectiveness (E)

Univariate and bivariate analyses were conducted to assess the impact of the intervention on specified outcomes. Specifically, to what extent was the community-focused patient navigation intervention effective at producing the positive types of results obtained from previous patient navigation intervention efficacy studies? Analyses of primary outcomes involved comparison of the counts of patients’ pre-intervention and post-intervention barriers, with a focus on the overall robustness of barrier reduction at the participant level using repeated measures ANOVA. Analyses of secondary outcomes included comparisons of pre-post patient-reported outcomes of global physical health and global mental health (PROMIS), self-efficacy (PROMIS), patient satisfaction with medical services (PSQ-18), and patient satisfaction with navigator (PSNI-I) using paired t-tests, as well as examination of the robustness of these comparisons across participant subgroups. All analyses were conducted using the sample of patients who completed the intervention as well as the post-intervention questionnaires (*n* = 207).

#### RE-AIM: adoption (a)

The Adoption component of the evaluation assessed the extent to which the community-focused patient navigation program was utilized among individuals and clinical teams within the cancer center: (a) Descriptive analyses were used to evaluate the number of clinical referrals made by cancer center staff over the course of the intervention. The 39-month period of active enrollment was split evenly into three time periods (Year 1, Year 2, and Year 3). Percent change in the total number of referrals was evaluated over time and organized by staff specialty to evaluate levels of utilization and cumulative referral count was graphed in order to descriptively assess adoption over time; and (b) A qualitative mixed methods survey designed to assess staff perspectives on intervention uptake and acceptability was distributed to staff who had been invited to make referrals to the intervention.

#### RE-AIM: implementation (I)

Program implementation was evaluated through a multidimensional assessment that included (a) univariate analyses to assess the fidelity of implementation by specifically assessing the timeliness between referral and first patient contact, calculated as the percentage of intervention deliveries that adhered to this program’s expectation of three or fewer days; (b) process-level analyses of standardized documentation and technology systems (e.g., REDCap and manual EMR data checks), including necessary adaptations made during the course of the three-year implementation, to address consistency of implementation across time; and (c) retrospective quantification of intervention costs.

#### Maintenance (M)

The extent to which the program was able to become successfully maintained within the cancer care setting was evaluated through (a) direct assessment of whether the intervention was still ongoing 6 months post-study funding [46], and (b) process-level analyses of efforts taken, at the research team and setting levels, to identify adaptations made following completion of the intervention to ensure sustainability of the community-focused patient navigation intervention.

## Statistical analyses

Data stored in REDCap were exported in a de-identified format and imported into SPSS and R, where all statistical analyses were conducted. Patient-reported demographic and disease characteristics were analyzed using descriptive statistics associated with the Reach aim. Pre- and post-intervention barriers data and patient-reported outcomes were analyzed using univariate, bivariate, and repeated measures analyses in SPSS to address the Effectiveness aim. Clinical referral data were descriptively evaluated based on percent increase/decrease across the 3 years of the intervention, and qualitative data were analyzed by content analysis to determine the extent to which the Adoption aim was met. Analyses of the fidelity of the intervention, including timeliness, consistency, and costs, were documented as part of the Implementation aim. Finally, process-level descriptions, including the summation of weekly team meetings and direct communications with the Principal Investigator (Hamann) of the community-focused patient navigation intervention were used to assess the Maintenance aim. All graphics were produced in R.

## Results

### Reach: participant demographic and disease characteristics

**Fig. 1:**
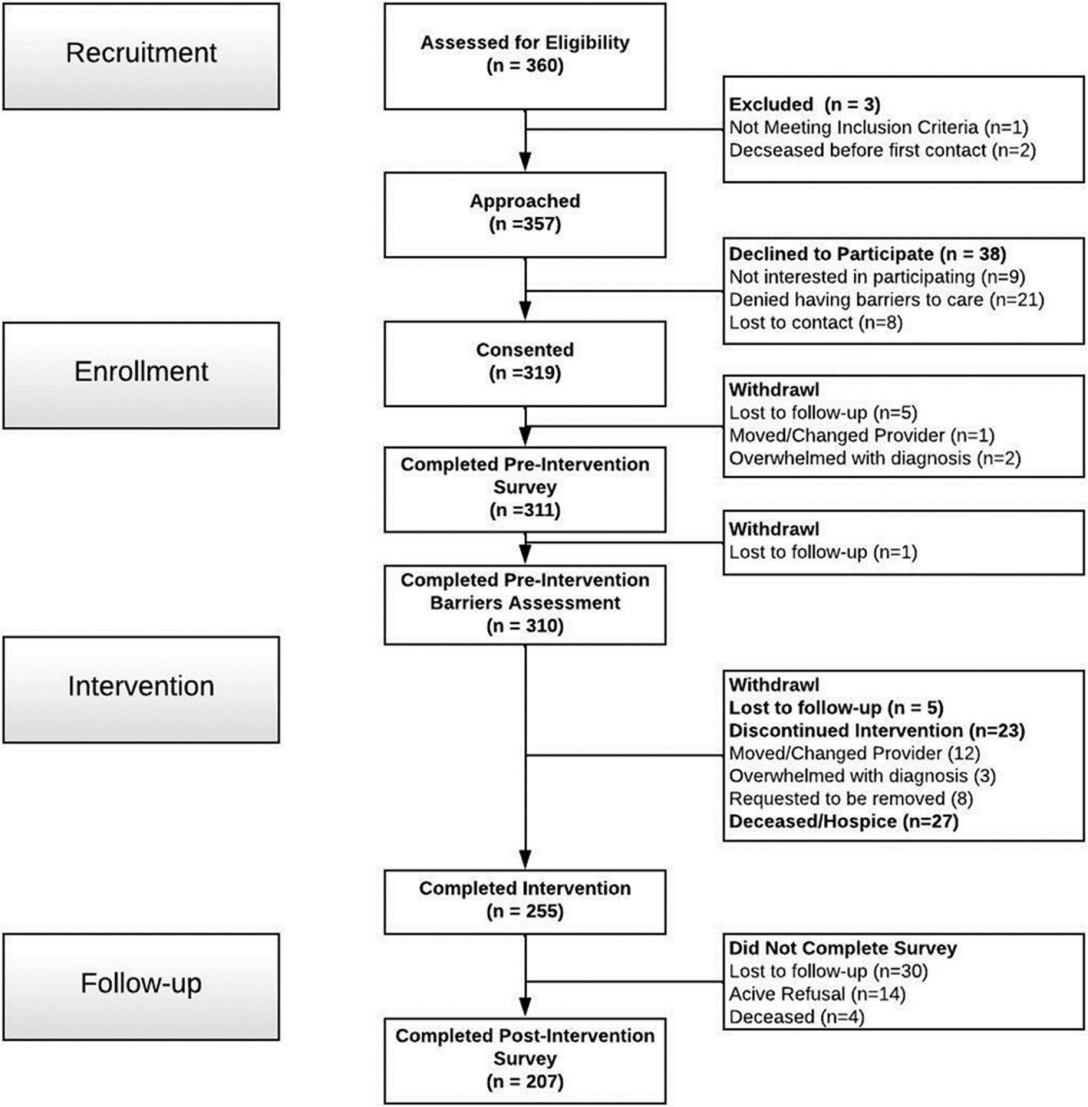
Community-Focused Patient Navigation CONSORT Diagram

Patients (*n* = 311) were enrolled regardless of cancer type or stage and were excluded only if they did not have a definitive diagnosis of cancer or if they died prior to first contact with the study team (*n* = 3; See CONSORT; Fig. 1). Descriptive analyses of the demographic characteristics of enrolled participants reflected a largely underserved population. The majority of participants self-identified as Hispanic/Latino, reported being enrolled in Medicaid, and reported household incomes of less than $35,000 per year (Table 2). A strong minority of patients indicated Spanish to be their primary language (41.2%) and reported experiencing housing insecurity (i.e., “worry or concern about not having stable housing”) within the past 6 months (41.2%). Based on zip code analysis, approximately 12 % of participants lived in areas designated by HRSA as ‘rural’ [49]. Examination of disease characteristics indicated that enrolled patients were most commonly diagnosed with gastrointestinal cancer and with late stage (Stage III or Stage IV) disease; approximately half had only recently been diagnosed with cancer or recently initiated cancer treatment (Table 2). The analyses of variance comparing demographic and disease characteristics among participants who enrolled in the patient navigation intervention (*n* = 311), participants who completed the intervention (*n* = 255), and participants who completed both the intervention and the post-intervention survey (*n* = 207) indicated overall comparability and no statistically significant differences (*p*’s > .05) (Table 2). Across the intervention, there was a 10% attrition rate due to mortality (See CONSORT; Fig. 1).

**Table 2:** Demographic and disease characteristics for enrolled, completed, and follow-up patients in community-focused patient navigation intervention

Demographic and Disease Characteristics	Enrolled (*n* = 311)	Completed Intervention (*n* = 255)	Completed Intervention + Survey Follow-Up (*n* = 207)
**Demographics**			
**Ethnicity**			
Hispanic/Latino	60.8% (*n* = 189)	62.0% (*n* = 158)	63.3% (*n* = 131)
Non- Hispanic/Latino	39.2% (*n* = 122)	38.0% (*n* = 97)	36.7% (*n* = 76)
**Race**			
White	90.7% (*n* = 282)	91.0% (*n* = 232)	92.3% (*n* = 191)
Other (Asian, Black, Native American)	9.3% (*n* = 29)	9.0% (*n* = 23)	7.7% (*n* = 16)
**Primary Language**			
Spanish	41.2% (*n* = 128)	40.8% (*n* = 104)	43.0% (*n* = 89)
English	56.9% (*n* = 177)	57.3% (*n* = 146)	54.6% (*n* = 113)
Other	1.2% (*n* = 6)	2.0% (*n* = 5)	2.4% (*n* = 5)
**Gender**			
Male	48.2% (*n* = 150)	48.9% (*n* = 117)	46.4% (*n* = 96)
Female	51.4% (*n* = 160)	53.7% (*n* = 137)	53.1% (*n* = 110)
Other	.3% (*n* = 1)	.4% (*n* = 1)	.5% (*n* = 1)
**Age**			
Under 65 yo	69.1% (*n* = 215)	72.9% (*n* = 186)	72.5% (*n* = 150)
Over 65 yo	28.6% (*n* = 89)	24.3% (*n* = 62)	24.2% (*n* = 50)
**Birth Country**			
United States	47.9% (*n* = 149)	47.8% (*n* = 122)	46.9% (*n* = 97)
Mexico	44.1% (*n* = 137)	43.9% (*n* = 112)	45.9% (*n* = 95)
Other	8.0% (*n* = 25)	8.2% (*n* = 21)	7.2% (*n* = 15)
**Marital Status**			
Married or Partnered	42.8% (*n* = 133)	43.9% (*n* = 112)	44.9% (*n* = 93)
Single	57.2% (*n* = 178)	56.1% (*n* = 143)	55.1% (*n* = 114)
**Geographic**			
Rural AZ	12.5% (*n* = 39)	12.2% (*n* = 31)	10.1% (*n* = 21)
Urban AZ	83.6% (*n* = 260)	85.9% (*n* = 219)	88.4% (*n* = 183)
Out of State	3.9% (*n* = 12)	2.0% (*n* = 5)	1.4% (*n* = 3)
**Highest Level of Education**			
High School or Less	63.7% (*n* = 198)	65.1% (*n* = 166)	68.1% (*n* = 141)
More than High School	42.8% (*n* = 113)	34.9% (*n* = 89)	31.9% (*n* = 66)
**Household Income**			
Less than $35,000/year	55.3% (*n* = 172)	56.9% (*n* = 145)	56.5% (*n* = 117)
More than $35,000/year	11.6% (*n* = 36)	12.9% (*n* = 33)	12.6% (*n* = 26)
Prefer not to answer	33.1% (*n* = 103)	30.2% (*n* = 77)	30.9% (*n* = 64)
**Insurance**			
Medicaid	56.3% (*n* = 175)	58.0% (*n* = 148)	58.9% (*n* = 122)
Not Medicaid	43.7% (*n* = 136)	42.0% (*n* = 107)	41.1% (*n* = 85)
**Home Ownership**			
Rent	47.6% (*n* = 148)	51.4% (*n* = 131)	53.6% (*n* = 111)
Own	30.2% (*n* = 94)	29.0% (*n* = 74)	28.0% (*n* = 58)
Other arrangement	22.1% (*n* = 69)	19.6% (*n* = 50)	18.4% (*n* = 38)
**Employment**			
Employed	22.2% (*n* = 69)	23.9% (*n* = 61)	25.1% (*n* = 52)
Unemployed	54.7% (*n* = 170)	56.9% (*n* = 145)	58.0% (*n* = 120)
Retired	23.2% (*n* = 72)	19.2% (*n* = 49)	16.9% (*n* = 35)
**Housing “worry” in past 6-months**			
Yes	41.2% (*n* = 128)	43.1% (*n* = 110)	43.0% (*n* = 89)
No	58.5% (*n* = 182)	56.5% (*n* = 144)	56.5% (*n* = 117)
**‘Underserved’ (racial/ethnic minority and/or insured through Medicaid or uninsured)**			
Yes	81.7% (*n* = 254)	83.9% (*n* = 214)	84.1% (*n* = 174)
No	18.3% (*n* = 57)	16.1% (*n* = 41)	15.9% (*n* = 33)
**Disease Characteristics**			
**Cancer Type**			
Breast	15.8% (*n* = 49)	16.5% (*n* = 42)	17.4% (*n* = 36)
Gastrointestinal	24.4% (*n* = 76)	23.9% (*n* = 61)	24.6% (*n* = 51)
Genitourinary	17.0% (*n* = 53)	17.6% (*n* = 45)	16.9% (*n* = 35)
Skin	3.5% (*n* = 11)	3.9% (*n* = 10)	3.9% (*n* = 8)
Lung	5.5% (*n* = 17)	4.7% (*n* = 12)	4.3% (*n* = 9)
Head and Neck	10.0% (*n* = 31)	10.6% (*n* = 27)	10.1% (*n* = 21)
Hematological	11.6% (*n* = 36)	12.2% (*n* = 31)	12.1% (*n* = 25)
Brain	1.6% (*n* = 5)	1.6% (*n* = 4)	1.4% (*n* = 3)
Other /Unknown	8.4% (*n* = 26)	7.1% (*n* = 18)	7.2% (*n* = 15)
**Cancer Stage**			
Early Stage (Stage 0, I, II)	27.3% (*n* = 85)	26.7% (*n* = 68)	28.0% (*n* = 58)
Late Stage (Stage III, IV)	49.5% (*n* = 154)	51.0% (*n* = 130)	49.8% (*n* = 103)
Other/Unknown	23.2% (*n* = 72)	22.4% (*n* = 57)	22.2% (*n* = 46)
**Cancer Care Continuum Status**			
Recent Diagnosis or Treatment Initiation	50.2% (*n* = 156)	49.4% (*n* = 126)	47.8% (*n* = 99)
Active Treatment Continuation	22.8% (*n* = 71)	24.0% (*n* = 61)	24.6% (*n* = 51)
Palliative Care	13.8% (*n* = 43)	13.7% (*n* = 35)	14.0% (*n* = 29)
Post-Treatment Survivorship	4.8% (*n* = 15)	4.7% (*n* = 12)	4.8% (*n* = 10)

### Reach: participant representativeness

For each of the 3 years of intervention implementation, cancer registry data were summarized to identify: (1) the size of the total patient population seen at the cancer center (*n* = 1943 patients in Year 1; *n* = 1937 patients in Year 2; and *n* = 2225 patients in Year 3), (2) the number of patients seen at the cancer center who met criteria for being ‘underserved’ (i.e., uninsured, on Medicaid, and/or Hispanic, Black, American Indian/Alaska Native, Native Hawaiian, Multiracial) (*n* = 375 patients in Year 1; *n* = 314 patients in Year 2; and *n* = 395 patients in Year 3), and (3) the percentage of the population of interest (i.e., underserved patients at the cancer center) that the community-focused patient navigation intervention was able to reach (19.7% of patients in Year 1; 30.6% of patients in Year 2; and 21.3% of patients in Year 3) (Table 3). These comparative ratios indicate that 82% of enrolled participants in the community-focused patient navigation intervention were ‘underserved,’ thus the intervention reached approximately 23% of the total population of interest (i.e., total number of underserved patients seen at the cancer center) over the 3 years of enrollment.

**Table 3:** Representativeness and proportion of patients that participated in the community-focused patient navigation intervention

REACH	Intervention (*n*)	Cancer Center (*n*)	Percent of population of interest (%)
**Year 1**			
Total Number of Patients	85	1943	4.4%
Number of Underserved Patients • Racial/Ethnic Minority and/or • Medicaid/Uninsured	74	375	19.7%
**Year 2**			
Total Number of Patients	120	1937	6.2%
Number of Underserved Patients • Racial/Ethnic Minority and/or • Medicaid/Uninsured	96	314	30.6%
**Year 3**			
Total Number of Patients	106	2225	4.8%
Number of Underserved Patients • Racial/Ethnic Minority and/or • Medicaid/Uninsured	84	395	21.3%

The number of people and percentage of total cancer patient population enrolled in the intervention between June 2018 and October 2021 (Year 1 = 1st 13 months; Year 2 = 2nd 13 months; Year 3 = 3rd 13 months). Population of interest for the community-focused patient navigation intervention was ‘underserved’ patients with cancer defined as a racial/ethnic minority and/or insured through Medicaid or uninsured

### Effectiveness: reducing barriers to cancer care

**Fig. 2:**
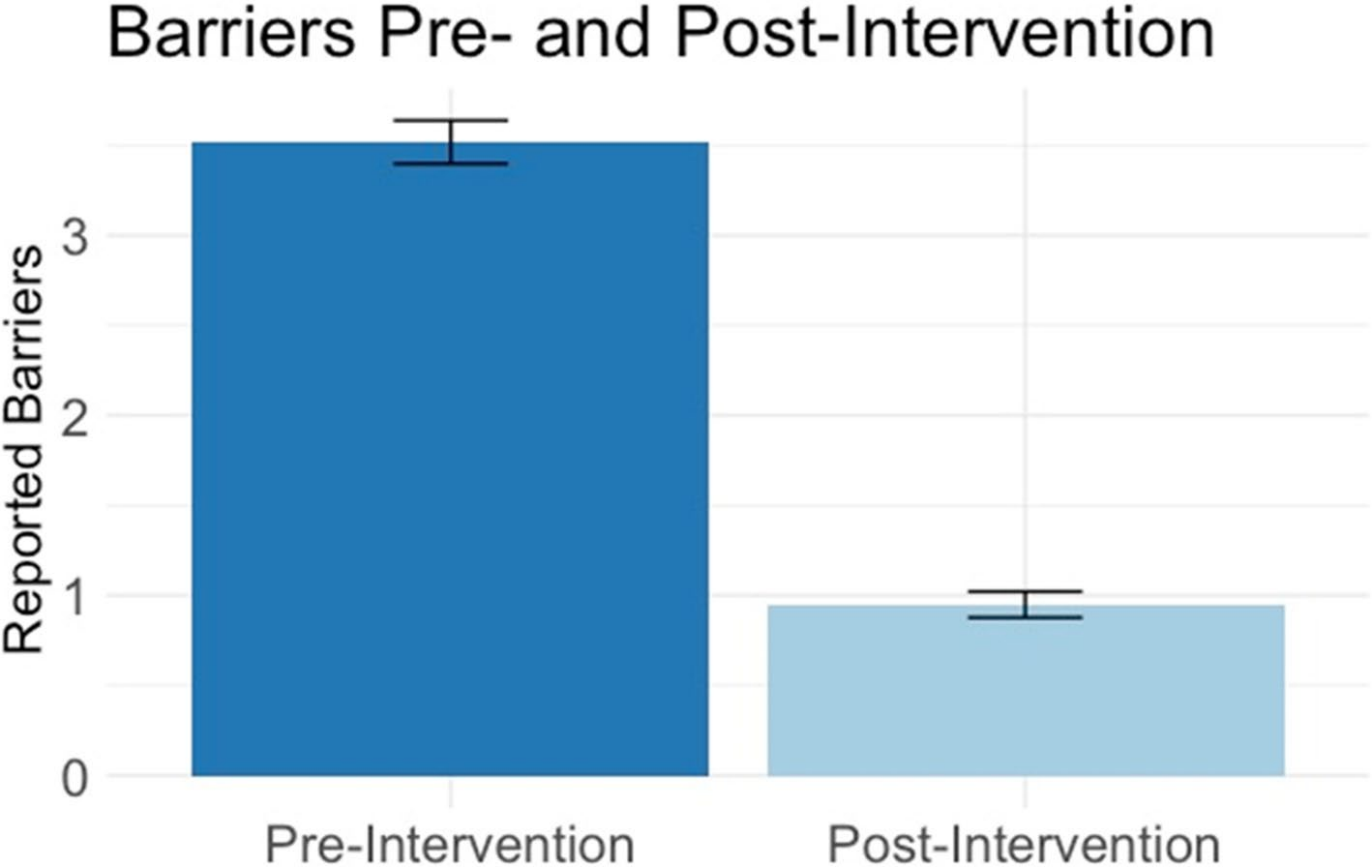
Primary Effectiveness Outcome: Average Barrier Count Per Participant at Pre-Intervention and Post-Intervention. At pre-intervention, participants reported, on average, 3.44 barriers to cancer care (dark blue). At post-intervention, participants had, on average, 0.94 barriers to cancer that were unresolved or unaddressed (light blue)

The primary effectiveness outcome of the community-focused patient navigation intervention was operationalized as the percentage of pre-intervention barriers that were adequately addressed, per patient, by the community-focused navigator over the course of the 3-month intervention. Number of barriers reported at pre-intervention did not statistically differ between those who completed the intervention (*n* = 207) and those who did not (*n* = 104); *t*(310) = − 1.33, *p* = .159. The average number of pre-intervention patient-reported barriers for the 207 participants who completed the 3-month intervention and the post-intervention survey was 3.54 (range: 1–10). Examination of endorsement frequency indicated that the 10 most prevalent patient-reported barriers to cancer care (in order of descending frequency) included: (1) can’t afford utilities, (2) needs vision care, (3) can’t afford housing, (4) public transportation not readily available, (5) no health insurance, (6) can’t afford co-pay/deductible, (7) no primary care provider, (8) needs hearing test, (9) feels depressed, and (10) feels overwhelmed by paperwork. At the time of the post-intervention assessment, their average number of unresolved barriers was 0.94 (range: 0–7) with an average of 74.7% of each patients’ pre-intervention barriers being either adequately addressed (i.e., a resource was provided, although the barrier may not have been completely resolved) or fully resolved (i.e., a resource was provided, and the barrier was resolved) (Fig. 2). Number of reported pre-intervention barriers did not differ by participant age, cancer stage, or status along the cancer care continuum but did differ based on intervention year, r(207) = −.227, *p* < .001. Therefore, a repeated-measures ANOVA accounting for a covariate of intervention year (Year 1, Year 2, or Year 3) indicated that barrier number significantly decreased between the initiation (i.e., pre-intervention) and completion (i.e., post-intervention) of the community-focused patient navigation intervention, F(1,207) = 117.62, *p* < .001, as well as a large effect size, .365. The interaction between intervention year and barrier was not significant (*p* = .061). The two most common actions taken by the navigator to address a patient-reported barrier were to provide a resource to the patient and to contact a resource on behalf of the patient.

### Effectiveness: improvement in patient-reported outcomes

**Fig. 3:**
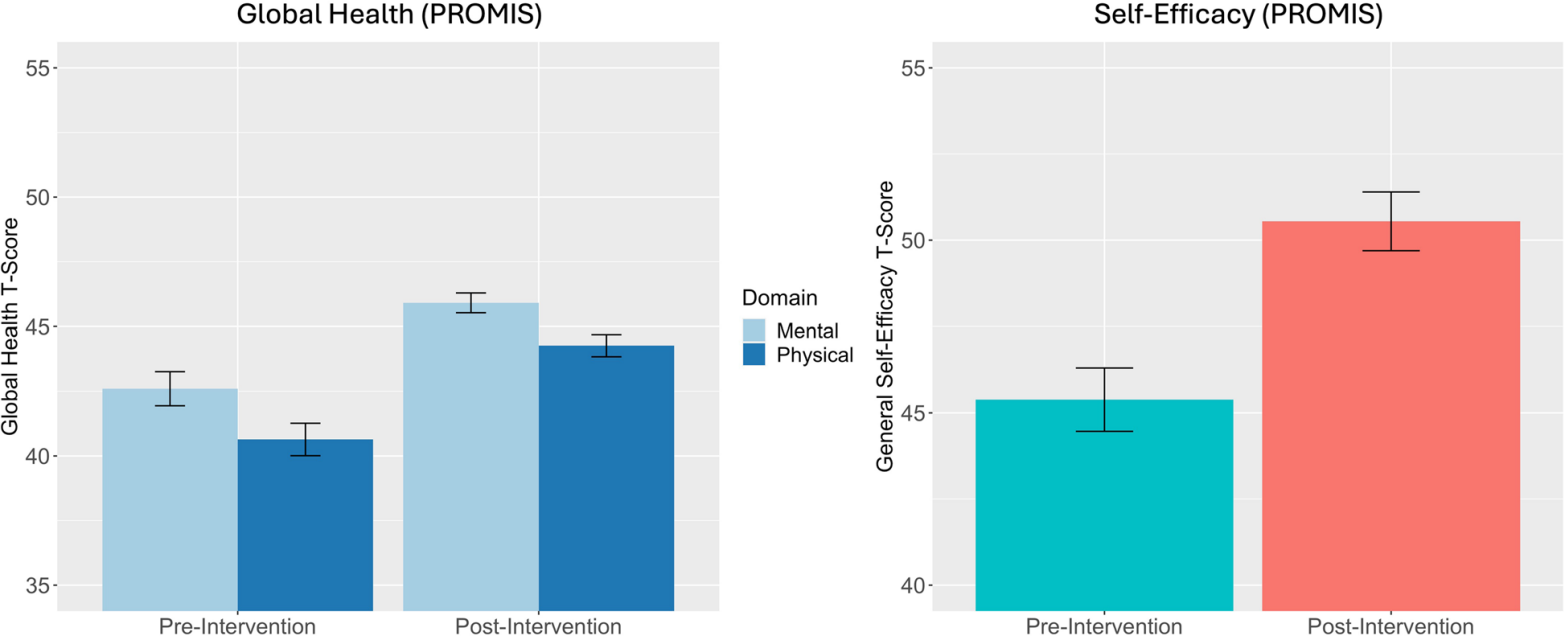
Secondary Effectiveness Outcomes: Patient-Reported Outcomes at Pre-Intervention and Post-Intervention. Participants demonstrated significant improvement in their global mental health between pre- and post-intervention, *t*(205) = −3.810, *p* < .001; *d* = −.265. Participants demonstrated a significant increase in their global physical health between pre- and post-intervention, *t*(205) = −6.004, *p* < .001; *d* = −.418. Participants demonstrated a significant increase in self-efficacy between pre- and post-intervention, *t*(205) = −5.321, *p* < .001; *d* = −.371

To assess the impact of the community-focused patient navigation intervention on patient-reported outcomes, paired-sample t-tests were conducted on patients’ pre-intervention and post-intervention questionnaires. As indicated in Fig. 3, participants (*n* = 207) exhibited significant improvement in their global mental health after completing the intervention (*M* = 45.93, *SD* = 5.5) compared to before the intervention (*M* = 42.64, *SD* = 9.4) and this improvement, − 3.3, 95%CI [− 5.0, − 1.6], was statistically significant, *t*(205) = − 3.810, *p* < .001; *d* = −.265. Similarly, participants exhibited significant improvement in their global physical health after completing the intervention (*M* = 44.3, *SD* = 6.1) compared to before the intervention (*M* = 40.7, *SD* = 9.0), and this improvement, − 3.7, 95%CI [− 4.9, − 2.5], was statistically significant, *t*(205) = − 6.004, *p* < .001; *d* = −.418. There was also significant improvement in patient-reported self-efficacy such that participants’ scores after completing the intervention (*M* = 50.55, *SD* = 12.23) were higher than their scores before the intervention (*M* = 45.38, *SD* = 13.19) and this improvement, − 5.16, 95%CI [− 7.1, − 3.3], was statistically significant, *t*(205) = − 5.321, *p* < .001; *d* = −.371.

Following the intervention, participants (*n* = 207) also demonstrated overall increases in their satisfaction with their medical services, including patient reports of significantly greater general satisfaction, greater satisfaction with their communication with their medical teams, improvement in financial aspects of their medical care, greater amount of time that their doctors spent with them, and greater accessibility and convenience related to their medical care (Table 4). Following completion of the intervention, patients also reported strong satisfaction with the community-focused patient navigator, the navigator’s efforts to resolve their barriers, and the interpersonal relationships they established over the course of the intervention as evidenced by an average score of 40.03 (*SD* = 6.2) on the Patient Satisfaction with Navigator Interpersonal Relationship Scale, a score consistent with previous interventions that included effective navigators [45].

**Table 4:** Pre- and Post-Intervention Patient Satisfaction Questionnaire (PSQ-18)

	Mean	Std Dev	S.E. mean	Paired *t* Test		
Patient Satisfaction Questionnaire (PSQ-18)				*t* value	df	Sig (two-tailed)
**General Satisfaction**						
Pre-Intervention	3.88	1.11	.08	−2.12	206	.035*
Post-Intervention	4.05					
**Interpersonal Manner**						
Pre-Intervention	4.03	1.03	.07	−1.35	206	.178
Post-Intervention	4.13					
**Communication**						
Pre-Intervention	4.08	.92	.06	−2.20	206	.029*
Post-Intervention	4.22					
**Financial**						
Pre-Intervention	2.66	1.45	.10	−7.69	206	<.001**
Post-Intervention	3.43					
**Time with Doctor**						
Pre-Intervention	3.82	1.18	.08	−2.68	206	<.001**
Post-Intervention	4.04					
**Accessibility and Convenience**						
Pre-Intervention	3.57	1.03	.07	−4.81	206	<.001**
Post-Intervention	3.91					

Note: *p < .05 and **p <.001

### Adoption: quantitative assessment of staff-level engagement in intervention

Prior to implementing the community-focused patient navigation intervention, the research team evaluated the existing clinical flow of supportive care referrals at the cancer center. Informal communication with the social work team, a palliative care physician, and a manager of the nurse navigators revealed that nurse navigators were typically the first members of the clinical care team to identify patient’s barriers to cancer care in advance of their initial appointments with their oncologists. Following treatment initiation and at further points along the cancer care continuum, social workers tended to receive the majority of referrals for assistance with patients’ barriers to care and requests for supportive care services. Based on this preliminary assessment, our research team invited members of all clinical care teams to participate in the patient navigation intervention (i.e., social workers, nurse navigators, financial counselors, physicians, and clinical research coordinators), but placed primary focus for staff engagement efforts on the social worker and nurse navigator teams.

**Fig. 4:**
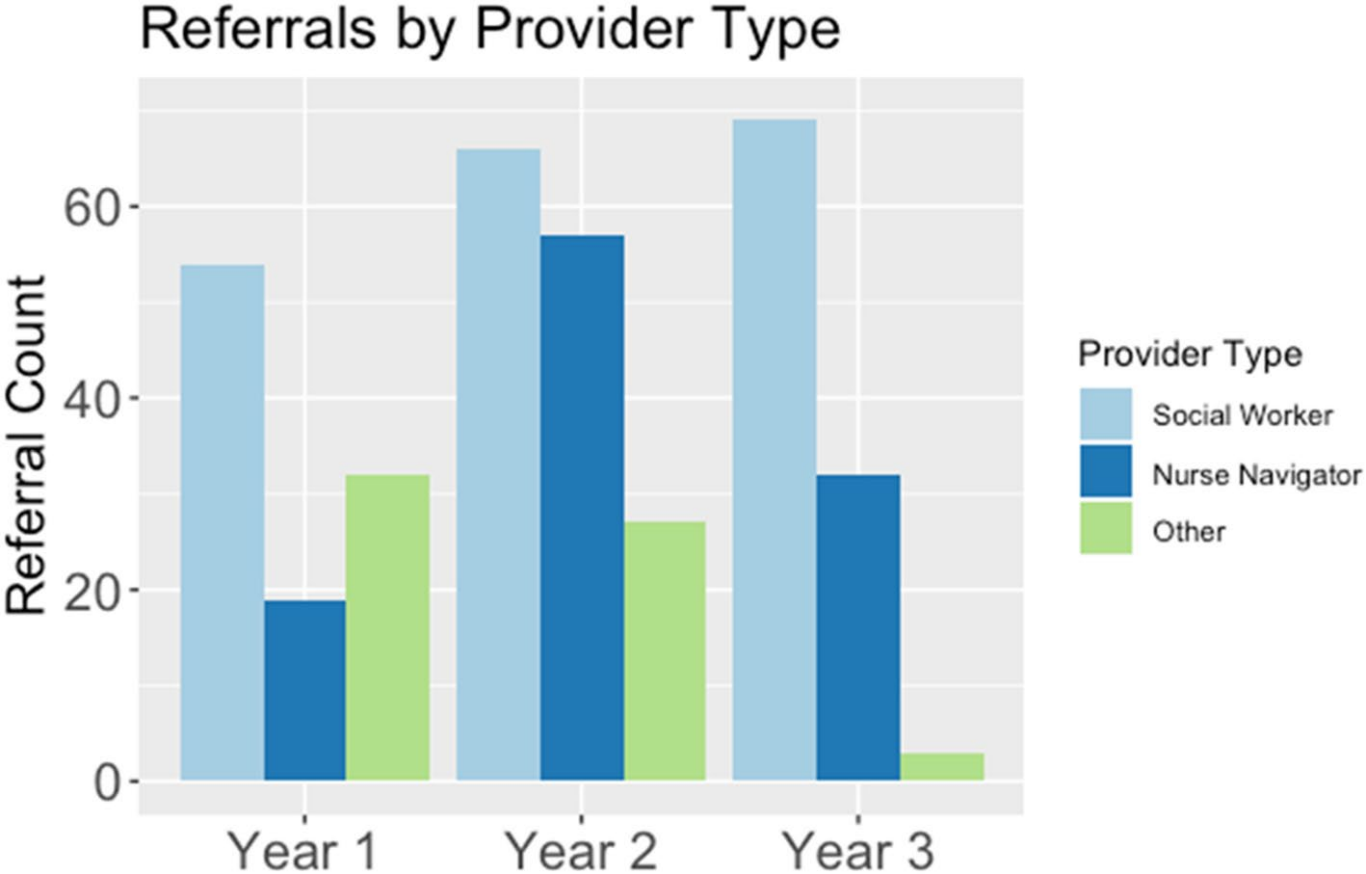
Adoption: Referrals Over Time by Specialty Provider Type. The number of referrals to the community-focused patient navigation intervention were organized across the 3 years of the intervention between June 2018 and October 2021 (Year 1 = 1st 13 months; Year 2 = 2nd 13 months; Year 3 = 3rd 13 months). Referrals to the community-focused patient navigation intervention are organized by provider type (social worker, nurse navigator, or other provider)

**Fig. 5:**
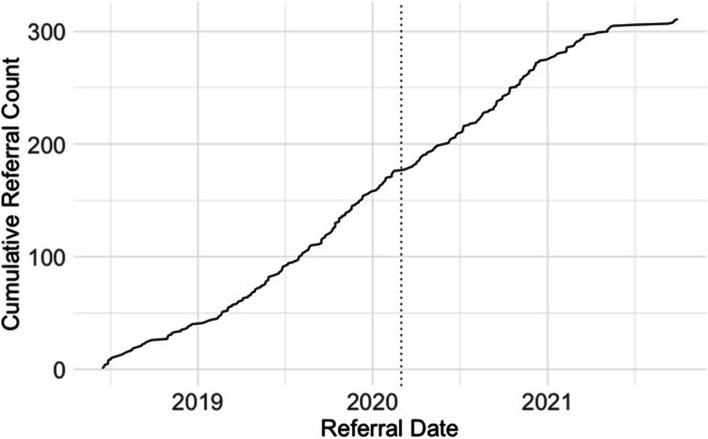
Cumulative Referral Count by Referral Date. The number of referrals to the community-focused patient navigation intervention are represented as a cumulative count across the duration of the intervention between June 2018 and October 2021. The solid line begins with the first patient referral (June 15, 2018) and concludes with the final patient referral (September 30, 2021). The dashed line indicates the start of COVID-19 (March 1, 2020)

Adoption of the intervention by staff at the cancer center was assessed quantitatively in terms of how referral rates across provider specialties (e.g., social worker, nurse navigator, financial specialist, etc.,) changed over the course of the 3-year intervention. The community-focused patient navigation intervention received a total of 360 referrals across the 3-year period. This included 189 referrals from social workers, 108 referrals from nurse navigators, and 63 referrals from members of other clinical specialty teams (3 from physicians, 10 from financial specialists, and 50 from clinical research team members). Percent increase in the number of referrals made to the patient navigation intervention was operationalized as a metric to reflect the increasing strength of adoption across the course of intervention implementation. Results indicated a 42.9% increase in the number of referrals between Year 1 (105 referrals) and Year 2 (150 referrals); a 1% decrease in the number of referrals between Year 1 and Year 3 (104 referrals); and a 44.2% decrease in the number of referrals between Year 2 and Year 3 (Fig. 4). Evidence of a decrease in referrals in Year 3 was likely due to both the onset of the COVID-19 pandemic in March 2020 and changes in intervention enrollment capacity associated with the anticipated end of the intervention in Fall 2021 (Fig. 5). Within clinical provider specialties, referrals from the social work team increased 27.8% between Year 1 (54 referrals) and Year 3 (69 referrals) and referrals from the nurse navigation team increased 68.4% between Year 1 (19 referrals) and Year 3 (32 referrals), suggesting increasingly wider adoption of the community-focused patient navigation intervention into the clinical flow for the two clinical teams that were primarily responsible for managing supportive care referrals.

### Adoption: qualitative assessment of staff-level engagement in intervention

Qualitative methods were used to assess staff perceptions of the community-focused patient navigation intervention and the utility of the community-focused patient navigator’s care coordination efforts within the cancer center setting. Sixteen key cancer center clinical staff members were asked to complete an anonymous feedback survey at the conclusion of the study. Eight staff members responded to the survey including three nurse navigators, three social workers, one nurse navigator manager, and one care coordinator. Overall, clinical staff reported being “very satisfied” with the community-focused patient navigation program. The majority of respondents (*n* = 7) indicated that they had submitted a minimum of 20 referrals to the community-focused patient navigation program, with two respondents reporting that they had submitted over 50 referrals. When asked to write free responses to support their satisfaction endorsement, several adoption-related themes emerged that facilitated the navigator’s integration with cancer center staff including the importance of bilingual delivery of service, a strong connection to and knowledge of community resources, and the ability to take initiative quickly. Barriers to intervention adoption included a mismatch between research related goals (e.g., study enrollment requirements such as use of patient consent form and use of baseline patient-reported questionnaires) versus clinical expectations (i.e., being able to receive a referral and quickly start working with a patient to address barriers to care). In addition, separation of the setting’s clinical delivery organization (i.e., Non-Profit Health System) and the research enterprise (Public university) [33] was identified as a barrier in the intervention delivery (e.g., regulatory challenges and EMR accessibility).

### Implementation: timeliness of intervention delivery

The fidelity of an intervention includes an assessment of the timeliness with which the intervention was implemented. The community-focused patient navigator was instructed to act quickly (modeling as close to a “warm handoff” as possible) following receipt of a referral, to connect with the patient (in person or by phone), to explain the intervention and answer any questions the patient might have, and to invite the patient to participate in the intervention. We calculated the number of days between patient referral and date of first contact, and found the average to be 2.66 days (range: 1–35; *SD* = 5.39). Setting a maximum threshold of 3 days, we found that the community-focused patient navigator successfully met the threshold criteria 76.4% (*n* = 275) of the time, suggesting that the majority of intervention initiations were delivered in a timely manner.

### Implementation: intervention consistency

Aligned with the objective of intervention fidelity, all intervention activities were tracked through REDCap, a secure web application for managing electronic databases. These data provided an opportunity for assessment of the extent to which intervention procedures were adhered to consistently and in the expected manner. Following the longitudinal project design, REDCap automatically delivered email reminders to the community-focused navigator and to the primary study coordinator to ensure fidelity of intervention components including completion of baseline surveys, two-month barrier assessment check-in phone calls, and 3-month post-intervention phone calls. Although an email reminder does not guarantee follow-through, this feature contributed to consistency of implementation across participants over the course of the 3-year intervention. For example, the email reminder for the two-month barrier assessment check-in phone call was automated to be sent to the Navigator and study project coordinator exactly 62 days after patient’s consent date. Of the 255 participants who completed the intervention (i.e., remained in the intervention for a total of 3 months), 237 participants (93%) received a two-month barrier assessment check-in phone call and/or had a documented reason for not being contacted (e.g., participant had passed away). The average number of days between the consent date and the date the actual two-month barrier assessment check-in phone call was completed was 62.2 (range: 28 days – 139 days; standard deviation: 14.8 days), suggesting close adherence to the automated schedule.

Throughout the 3-year intervention, significant adaptations in the structure of intervention delivery did not occur. Minor adaptations to the protocol were considered by the study team at weekly meetings, and were sometimes implemented. For example, within the first 6 months of intervention implementation, the navigator indicated that she had greater success at communicating with patients outside of the standard 9–5 work hours, so adjustments were made to her work schedule to accommodate after-hours contact.

**Fig. 6:**
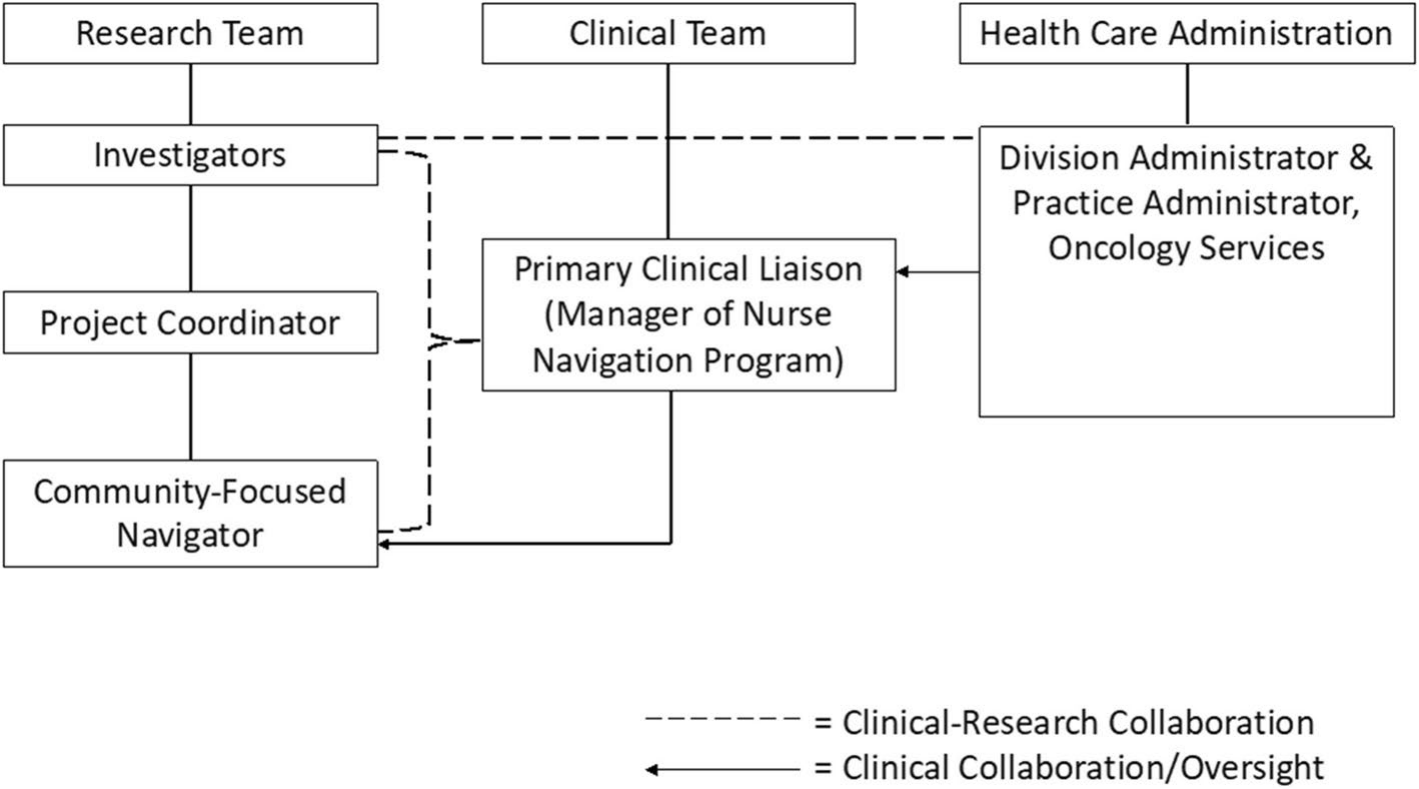
Clinical Research Partnership of the Community-Focused Patient Navigation Intervention. This diagram depicts the structure of clinical-research partnerships within the community-focused patient navigation intervention. Clinical-Research collaboration refers to members of the research or clinical team who were directly involved in day-to-day operations of the intervention. Clinical Collaboration/Oversight refers to members of the clinical or health care administrative teams who were indirectly involved in the intervention

The study’s research-clinical partnerships (See Fig. 6) promoted consistency of intervention implementation by maintaining regularly scheduled meetings and building consistently open lines of communication for intervention delivery. Specifically, the study team (i.e., investigators, project coordinator, and community-focused navigator) met weekly to review participant accrual and to discuss any database-related challenges. The study team and the primary clinical liaison (i.e., manager of nurse navigation program at the cancer center) met bi-weekly to discuss any clinically relevant questions (e.g., clarifying the categorization of a particular cancer type) and to review patient referral processes. The primary clinical liaison also provided direct supervision and clinical support for the community-focused navigator throughout the intervention. Biannually, the study investigators met with cancer center administrators to review intervention-related outcomes (e.g., patient-reported satisfaction with their medical care) and cost-effectiveness aspects of the intervention. These research-clinical partnerships remained strong and consistent across the course of the intervention’s implementation.

### Implementation: intervention costs

Evaluation of intervention costs was modeled off of an existing Patient Navigation Cost Framework [50], summarized within overarching categories of 1) Clinical Service Delivery Cost, 2) Maintenance of Research Infrastructure Cost, and 3) Clinical Partnership Cost (Table 5). Personnel represented the primary cost of delivering the intervention (i.e., Clinical Service Delivery Cost); space and supply costs were kept relatively minimal due to contributions made, in kind, by the intervention’s Principal Investigator within the cancer center.

**Table 5:** Estimated community-focused patient navigation program costs

Cost Categories	# Years	Estimate Amount ($)
**Clinical Service Delivery**		
Community-Focused Navigator Salary	4	$160,000 (= $40,000 × 4)
Community-Focused Navigator Fringe Benefits (~ 32%)	4	$51,200 (=$12,800 × 4)
Project Coordinator Salary	5	.25 FTE × 5
Investigator	5	.1 FTE × 5
**Maintenance of Research Infrastructure**		
Patient Materials (flyers, consent forms, business cards)	3.5	$500
Navigator Phone, Computer, Printer iPad	3.5	$10,000
Office Supplies (postage, folders, binders)	3.5	$1000
Mobile telephone costs	3.5	$1800
Database maintenance	3	In Kind Contribution
**Clinical Partnership**		
Office Space & Furnishings	5	In Kind Contribution
Clinical liaison	5	In Kind Contribution
Clinical Supervision/Support for Navigator	5	In Kind Contribution

### Maintenance: status of intervention 6 months Post-study funding

At the setting level, the Clinical Service Delivery (i.e., 1 FTE, lay patient navigator) of the community-focused patient navigation intervention has been maintained following the conclusion of the intervention study. Specifically, the study’s community-focused patient navigator was hired by the cancer center as a full-time employee in 2021 and has remained present and integrated within the cancer center’s nurse navigation program, receiving approximately 25 referrals per week. As part of this transition, the clinical service provider took over full salary support for the study’s community-focused patient navigator. Notably, the clinical service provider’s support did not include protected time for the community-focused patient navigator to continue maintenance of the study’s research infrastructure (e.g., database maintenance) for ongoing data collection. Thus, while maintenance of this intervention was achieved at the setting-level, the maintenance – at the individual patient level – cannot be adequately assessed because long-term patient-level follow-up was not conducted.

### Maintenance: process-level description of program sustainability

The transition from grant funding to institution funding by the clinical partner in December 2021 represented overall maintenance success. Planning for this objective had been initiated by the Principal Investigator (Hamann) and Co-Investigators (Calhoun, Armin, and Ali-Akbarian) in 2018, and included frequent routine contacts as well as scheduled biannual meetings between study investigators and clinical care administrators. The primary objectives of the biannual meetings were to 1) increase care coordination within the network of community clinics, and 2) establish the value of the community-focused patient navigation intervention for the clinical care system. Specifically, the study team aimed to provide evidence to administrators of the value of having a bilingual and bicultural navigator embedded within the supportive care team to provide necessary support for Spanish-only speaking patients and to provide culturally appropriate community resources to address underserved patients’ barriers to cancer care.

Once the community-focused patient navigation program was incorporated into the nurse navigator program within the cancer center clinical care team, the navigator worked with administrators and supportive care staff to distinguish her role and responsibilities from those of other established team members. For example, it was necessary to differentiate the navigator’s role in finding financial resources for patients from the role of the financial counselor, who specialized in insurance and financial payment plans. The community-focused patient navigator continued to receive referrals from clinical teams (primarily social work and nurse navigation) and continued to utilize a comprehensive barriers assessment. However, necessary adaptations to the program were also made: The patient navigator was no longer involved in consenting patients, collecting patient-reported outcome data, or tracking barrier reduction efforts and participant communications on REDCap.

Following conclusion of the intervention, the PI (Hamann) and research assistant (Ver Hoeve) conducted an informal, semi-structured interview with two lead cancer center administrators who were directly involved in the hiring of the community-focused patient navigator into the cancer center’s supportive care team. The administrators identified facilitating factors that supported the hiring process including the existence of a previously established FTE for a lay navigator at the cancer center institution, recognition that the community-focused patient navigation grant program “checked a lot of boxes” for what the cancer center was looking to improve upon, and perception that the continued presence of the patient navigator was fully supported by current supportive care staff.

## Discussion

The RE-AIM model was utilized to guide the implementation evaluation of an evidence-based, community-focused patient navigation intervention, with a focus on health equity in a new setting at an NCI-designated cancer center in the Southwestern United States. The implementation effectiveness of this three-year effort was demonstrated by the intervention’s ability to reach (‘R’) the population of underserved patients with cancer, effectively (‘E’) reduce barriers to cancer care while enhancing patient-reported outcomes, gain adoption (‘A’) among cancer center staff, be implemented (‘I’) with fidelity and consistency while maintaining costs, and ultimately maintain (‘M’) sustainability by successfully transitioning from a grant-funded intervention into an institution-funded community-focused patient navigation program.

To deliver on its maximum potential of reducing cancer health disparities, a patient navigation program must ensure that it focuses its efforts primarily on reaching historically medically underserved patients who carry the greatest cancer care burden. Approximately 40% of the population within the catchment area of this NCI-designated cancer center identifies as Hispanic [51], and is characterized as living in poverty (over 25%) or being uninsured (15%) [52]. The results of our implementation study indicate that 82% of the enrolled patients in our community-focused patient navigation intervention met the defined criteria for being ‘underserved’ and, over the three-year intervention period, the community-focused patient navigation intervention reached (i.e., enrolled) 23% of the total number of ‘underserved’ patients seen at the cancer center. Taken together, the utilization of an implementation science framework, particularly the use of RE-AIM’s ‘reach’ metric, facilitated an enhanced health equity focus by documenting the navigation intervention’s successful efforts at reaching and enrolling a representative sample of the medically underserved population of interest.

This implementation science study achieved effectiveness outcomes similar to those reported in prior patient navigation interventions [53–56]. Patients experienced significant reductions in their reported barriers to cancer care as well as significant improvements in their patient-reported outcomes, including their physical health, mental health, self-efficacy, and satisfaction with their medical care. Quality of life improvements among a primarily Hispanic participant sample represents a particularly meaningful outcome in light of a recent review suggesting that Hispanic patients with cancer often experience a lower quality of life (QoL) within the domains of psychological, physical, and social well-being [57]. In addition, robust improvements in patient satisfaction, including satisfaction with financial aspects of their medical care, suggest that the 3-month intervention may have been particularly useful for patients experiencing financial challenges. Notably, although some of these outcomes (e.g., patient satisfaction, quality of life, etc.,) have been previously demonstrated [21], our program’s inclusive approach regarding enrollment of participants with diverse cancer stage, cancer type, and status along the cancer care continuum further strengthens and expands the existing patient-report literature that uses patient navigation within an oncology setting. Taken together, this community-focused patient navigation intervention effectively demonstrated the expected result of barrier reduction and improved patient-reported health outcomes, and also highlighted patient-reported satisfaction with medical care (e.g., communication with doctor, time spent with doctor, financial distress, etc.) as a potentially valuable metric associated with healthcare quality [56, 58].

By tracking the number of referrals as a metric aligned with assessing the adoption of the community-focused patient navigation intervention at the staff-level, this study found that approximately 53% of referrals were initiated by the social work team, 30% were initiated by the nurse navigation team, and the remaining 17% were initiated by other clinical care providers. An increase of 43% in the number of referrals between the first and second year of the intervention indicated substantial adoption of the community-focused patient navigation intervention into the clinical care flow, particularly for the social work and nurse navigation teams who were primarily responsible for managing supportive care referrals as part of the cancer center’s standard procedures. However, a decrease in the number of referrals between Year 2 and Year 3 was also evident. This decrease coincided with the onset of the COVID-19 pandemic, at a time before vaccines were accessible, and represented not only a challenge for longitudinal research but also an indication of shifting clinical priorities as necessity required immediate responsiveness to the pandemic by all medical personnel, a powerful shift experienced by multiple clinical trials and research teams across the United States [59]. Recruitment declined slightly during the pandemic, and the work of the community-focused patient navigator transitioned from largely in-person (at the cancer center) to entirely remote, complicating the navigator’s ability to connect with patients who lacked consistent access to phone or internet services. Despite the pandemic, however, results of the mixed methods survey confirmed that, clinical care providers were clearly motivated to make referrals to this community-focused patient navigator, whom they generally viewed as someone with unique linguistic and cultural abilities who worked effectively with patients to reduce their barriers to care.

Despite previous efforts [23] and strong motivation [19] to formalize a business case for the incorporation of community-focused patient navigation into clinical cancer care, the sustainability of health equity-focused navigation programs remains a significant challenge [60, 61] and standardized navigation metrics on program implementation and sustainability are needed [62]. A critical process-level component of the present study was our description of intervention maintenance and how our research-clinical partnerships envisioned – from the outset of the study – a goal of transitioning the community-focused patient navigation intervention from a fully grant-funded project into a fully institutionalized cancer center program. Key strategies that supported this transition included routinely bringing clinic administrators into sustainability discussions throughout the intervention’s duration, effectively demonstrating the cultural and community value of the intervention in addressing the unmet needs within the cancer center’s catchment area, and providing convincing arguments on cost-effectiveness (e.g., showing benefit based on number of patients’ navigated to insurance coverage who subsequently initiated care at the cancer center). Our study team was also able to demonstrate consistency of this intervention’s delivery and fidelity through diligent use of REDCap’s data entry and reporting features which also supported our program sustainability goal. Importantly, however, the transition of our fully grant-funded community-focused patient navigation intervention into a fully institutionalized cancer center program did not include protected research time for ongoing data collection, a finding that appears consistent with the results of a recent national survey which identified a need for greater data collection among institutionally-funded patient navigation programs [62].

This study is not without limitations. The adoption component of RE-AIM recommends that a total number of staff (i.e., absolute number) within a designated setting be obtained to fully understand the percentage of staff that actually utilized the newly implemented intervention. We have provided estimated numbers of nurse navigators and social workers, but were unable to quantify the exact numbers of individual participants who sent referrals beyond their provider designation (e.g., social work, nurse navigation, clinical research coordinator). Further, there is no absolute number associated with other types of staff providers (e.g., doctors) because the research team loosely advertised the intervention to “any” clinical cancer care staff member who interacted with patients experiencing barriers to care. We also did not use a standardized measure to guide our assessment of intervention cost-effectiveness, although doing so might have strengthened our presentation of maintenance results and may have provided more guidance to other cancer centers looking to implement a community-focused patient navigation intervention. Additionally, our effectiveness outcomes (barrier reduction and patient-reported outcomes) could have been more meaningful if also associated with a clinical outcome (e.g., adherence) but this was not feasible within this study. Finally, although regular feedback was obtained from the navigator throughout the intervention (i.e., through weekly team meetings), the formal qualitative components of this implementation evaluation study did not include a direct perspective from the navigator (e.g., the navigator’s perspective on intervention adoption, delivery, and sustainability) was not documented in a way that directly aligned with RE-AIM processes. We received limited data from adoption survey responses and included only two semi-structured interviews assessing intervention sustainability factors only at the post-intervention time point. Thus, taken together, limited qualitative data within this intervention reduces scalability and generalizability of these findings.

This study provides an important contribution to the existing patient navigation and implementation science literature. First, to the best of our knowledge, it represents one of only a couple of published reports [63, 64] that uses the comprehensive implementation science evaluation framework, RE-AIM, to assess the implementation of a patient navigation program at a cancer care setting. By mapping our results onto the RE-AIM framework, we explicitly lay the groundwork for establishing the validity of these components of intervention implementation and strengthen the potential for building upon this type of patient navigation intervention to ultimately reduce cancer health disparities. Second, this study utilized patient navigation in patients experiencing more than 15 different types of cancer and at various stages along the cancer care continuum, including a significant proportion of patients with metastatic disease, as opposed to the bulk of patient navigation literature that focuses on early detection and diagnostic resolution. Third, this implementation science study included both quantitative and qualitative data to strengthen the depth of evaluation into the success of this intervention. Finally, the process-level strategies we identified when discussing the maintenance of our intervention represent an important contribution to the literature as the field struggles to establish the business case for community-focused patient navigation using funding provided directly by the cancer center institution. Taken together, this community-focused patient navigation intervention achieved successful implementation based on the RE-AIM metrics, demonstrated the use of implementation science to support improved health equity, and provided a description of processes to support transferability and scalability of patient navigation programs focused on reaching medically underserved patients with cancer.

## Conclusions

This research study used the implementation science evaluation framework, RE-AIM, to evaluate the implementation of a community-focused patient navigation program. The implementation effectiveness was demonstrated by the intervention’s ability to reach a population of underserved patients with cancer, effectively reduce barriers to cancer care while enhancing patient-reported outcomes, gain adoption among cancer center staff, be implemented with fidelity and consistency across time, and ultimately be maintained through transition from a grant-funded intervention into an institution-funded program. Program sustainability was achieved by routinely bringing clinic administrators into sustainability discussions throughout the intervention’s duration, by effectively demonstrating the cultural and community value of the intervention in addressing the unmet needs within the cancer center’s catchment area, and by demonstrating cost-effectiveness. These analyses indicate successful program implementation within a cancer care setting and lay the groundwork for establishing a standardized evaluation process for introducing and maintaining patient navigation programs focused on reaching and supporting underserved patients with cancer.

## Data Availability

The datasets analyzed during the current study are available from the corresponding author upon reasonable request.

## Abbreviations

CoC: Commisssion on cancer
NCI: National cancer institute
RE-AIM: Reach, effectiveness, adoption, implementation, and maintenance
FTE: Full-time equivalent
NIH: National institutes of health
EMR: Electronic medical record
QoL: Quality of life
